# A Modular Strategy to Prepare Multivalent Inhibitors of Prostate-Specific Membrane Antigen (PSMA)

**DOI:** 10.18632/oncotarget.415

**Published:** 2011-12-29

**Authors:** Sangeeta Ray Banerjee, Mrudula Pullambhatla, Hassan Shallal, Ala Lisok, Ronnie C. Mease, Martin G. Pomper

**Affiliations:** ^1^ The Russell H. Morgan Department of Radiology and Radiological Science, Johns Hopkins Medical School, Baltimore, MD

**Keywords:** Prostate-specific membrane antigen (PSMA), NAALADase, bivalent urea inhibitor, molecular imaging, multivalency, SPECT imaging

## Abstract

We have developed a modular scaffold for preparing high-affinity, homo-multivalent inhibitors of the prostate-specific membrane antigen (PSMA) for imaging and therapy of prostate cancer (PCa). Our system contains a lysine-based (∝-, ε-) dialkyne residue for incorporating a PSMA binding Lys-Glu urea motif exploiting click chemistry and a second lysine residue for subsequent modification with an imaging or therapeutic moiety. The utility of the multivalent scaffold was examined by synthesizing bivalent compounds 2 and 3 and comparing them with the monovalent analog 1. Determination of inhibition constants (*K*_i_) revealed that bivalent 2 (0.2 nM) and 3 (0.08 nM) are significantly more potent (~ 5 fold and ~ 11 fold, respectively) inhibitors of PSMA than monovalent 1 (0.9 nM). A single photon emission computed tomography (SPECT)-CT imaging study of [^111^In]3 demonstrated high and specific uptake in PSMA+ PC-3 PIP tumor until at least 48 h post-injection, with rapid clearance from non-target tissues, including kidney. A biodistribution study revealed that [^111^In]3 demonstrated 34.0 ± 7.5 percent injected dose per gram of tissue in PSMA+ tumor at 24 h post-injection and was capable of generating target-to-non-target ratios of ~ 379 in PSMA+ PC-3 PIP tumors *vs*. isogenic PSMA-negative PC3-flu tumors *in vivo*. The click chemistry approach affords a convenient strategy toward multivalent PSMA inhibitors of enhanced affinity and superior pharmacokinetics for imaging.

## INTRODUCTION

Prostate cancer (PCa) will kill an estimated 33,720 men in the US alone this year [1]. The integral membrane protein prostate-specific membrane antigen (PSMA) is becoming increasingly recognized as a viable target for imaging and therapy of prostate and other forms of cancer [2-4]. PSMA is significantly over-expressed in PCa and metastases, particularly with respect to the castration-resistant form [5]. Accordingly, PSMA may provide a negative prognostic indicator for PCa – enabling distinction of indolent from aggressive disease. Imaging PSMA has also provided insight into androgen signaling [6] and information on response to taxane therapy [7].

Recently we and others have demonstrated successful PSMA-targeted radionuclide imaging in experimental models of PCa using cysteine-glutamate or lysine-glutamate ureas. With those agents the radionuclide (^11^C, ^125^I, ^18^F) is attached to the cysteine or lysine moiety *via* a small prosthetic group [8-12]. For large molecular fragments, such as radiometal (^99m^Tc, ^68^Ga, ^111^In) chelators, organic fluorescent molecules, and nanoparticles, we have determined that a linking moiety of at least 20 Å (long-linker) between the large molecule and the lysine moiety facilitates productive binding [13-15]. We have also developed a PSMA-targeted, dual (radionuclide and optical) modality imaging platform that enables sequential, dual modality imaging [16]. As an extension of this program, here we prepare bivalent ligands with a view to improving the affinity and pharmacokinetic properties of the urea class of PSMA inhibitors. The strategy we employ can be generalized to multivalent compounds. Because they present multiple copies of the pharmacophore, multivalent ligands can bind to receptors with high avidity and affinity, thereby serving as powerful inhibitors [17, 18]. Various approaches have been reported to exploit multivalent scaffolds for the construction of molecular imaging probes [19-22]. However, the chemistry used to produce them can become complicated, even more so when a bifunctional chelator must be attached to a separately multimerized construct to introduce a radionuclide, for example, for imaging. Although, the concept of multimerization for PSMA targeted, near-infrared imaging agents has been proffered for *in vitro* cell binding studies [22], to our knowledge a multivalent PSMA-binding agent has not yet been shown to image PSMA successfully *in vivo*. Here we use click chemistry [23, 24] with our long-linker platform as a convenient route to build a modular scaffold for multimeric presentation of PSMA targeting species and demonstrate the enhanced ability of the bivalent form, over the corresponding monomer, to target PSMA *in vivo*.

## RESULTS AND DISCUSSION

**Chart 1:**
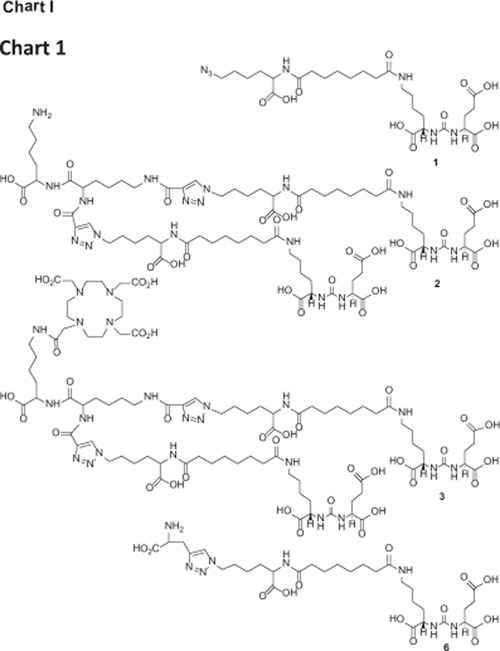


**Scheme 1:**
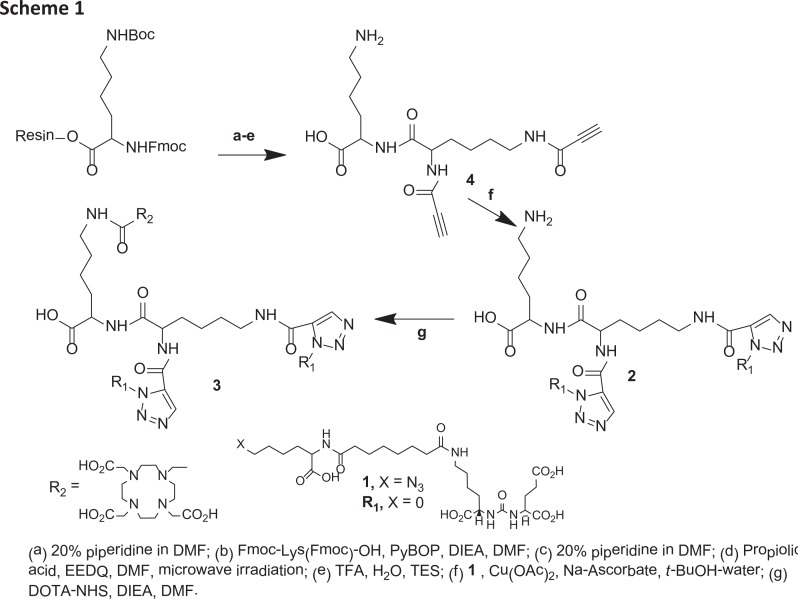


Our modular multivalent scaffold contains a lysine-based (∝-, ε-) dialkyne residue for incorporating PSMA binding Lys-Glu urea moieties exploiting click chemistry [23, 24] and a second lysine residue for subsequent modification with an imaging and/or therapeutic nuclide or a cytotoxic ligand for tumor cell kill. The divalent agent was anticipated to have a prolonged biological half-life and enhanced specific binding and retention in tissues expressing PSMA. To evaluate the anticipated multivalent effect, a versatile Lys-Glu-urea-based azide intermediate (1) was prepared to serve as a monovalent control compound (Chart 1) against the bivalent compound 2 and the DOTA-chelated bivalent urea analog, 3 to examine the effect of adding a chelating agent to bivalent urea 2. Compounds 2 and 3 were conveniently prepared by employing simple peptide coupling and click chemistry [23, 24] as shown in Scheme 1.

Starting with commercially available Fmoc-Lys(Boc)-Wang resin and using standard Fmoc-based solid phase peptide chemistry, 1 - 4 were prepared in suitable yields. In brief, Fmoc-Lys(Boc)-Wang was treated with 20% piperidine/DMF to remove the Fmoc group followed by coupling with commercially available Fmoc-Lys(Fmoc)-OH in the presence of benzotriazol-1-yloxy)tripyrrolidinophosphonium hexafluorophosphate (PyBOP) and N,N-diisopropylethylamine (DIEA). In the next step, a microwave-assisted coupling reaction was performed using propiolic acid in presence of 2-ethoxy-1-ethoxycarbonyl-1,2-dihydroquinoline (EEDQ) in DMF to improve the yield and purity of the desired product. Finally, 4 was isolated in ~ 40% yield after treating the resin with a cocktail of TFA/H_2_O/TES (95/2.5/2.5) at ambient temperature for 0.5 h. Compound 4, a Lys-based (∝-, ε-) dialkyne peptide, served as the key intermediate to introduce multimerization. Copper catalyzed click chemistry was employed using the azide intermediate 1 and dialkyne peptide 4 to produce multivalent compound 2 in moderate yield after purification by high-performance liquid chromatography (HPLC). Compound 3 was prepared by coupling the free amine of the lysine residue of 2 with the N-hydroxysuccinimide ester of DOTA-tris-acid using DIEA as a base in DMSO at ambient temperature for 4 h. Compound 3 was purified by HPLC and obtained in ~15% overall yield. Compound 3 was labeled with ^111^In at 95°C in 0.3 M NaOAc buffer within 20 min in ~70 - 90% yield and specific radioactivity of ~ 8.4 – 204.4 GB/μmol.

**Figure 1:**
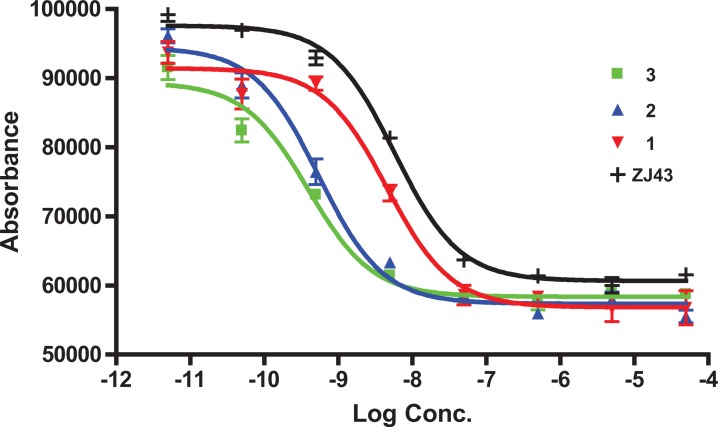
IC 50 curves of compounds 1-3 [8], [37]

PSMA inhibition constant (*K*_i_) values for 1 - 3 were determined using a fluorescence-based PSMA inhibition assay [8]. The data are presented in Table 1. As revealed from the *K*_i_ values, the binding affinity was found to increase 5-fold from monovalent 1 to bivalent 2. Interestingly, there was an 11-fold increase to the DOTA-chelated bivalent compound 3 compared to 1 leading to subnanomolar binding affinity for 3. Under the same experimental conditions, the *K*_i_ value of the known PSMA inhibitor ZJ-43 [25] was 1.16 nM, indicating the high inhibitory potency of 3. The inhibition curves of 1 - 3, which are expressed with respect to the amount of glutamate released from hydrolysis of the natural PSMA substrate, N-acetylaspartylglutamate (NAAG), are shown in Figure 1. A structurally similar triazole version of 1, compound 6 (Chart 1, Table 1) was also tested for PSMA inhibitory activity *in vitro* in a previous experiment [34]. The *K*_i_ value of 6 was 0.92 nM in that experiment, in which ZJ-43 demonstrated a *K*_i_ value of 0.35 (95% CI, 0.2 – 0.6 nM), suggesting that the affinity of 6 is likely significantly less than the bivalent compounds 2 or 3. Compound 6 was radiolabeled with ^99m^Tc and its biological properties were tested *in vivo* [34]. A manuscript describing those biological data is in preparation.

**Table 1:** PSMA inhibitory activity

Compound	Ki [nM]	95% CI of Ki
**1**	0.91	0.58 nM to 1.45 nM
**2**	0.10	0.07 nM to 0.16 nM
**3**	0.08	0.05 nM to 0.12 nM
**ZJ43**	1.16	0.92 nM to 1.46 nM
**6**	0.92*	0.06 nM to 12 nM

* Separate experiment (see text)

**Figure 2:**
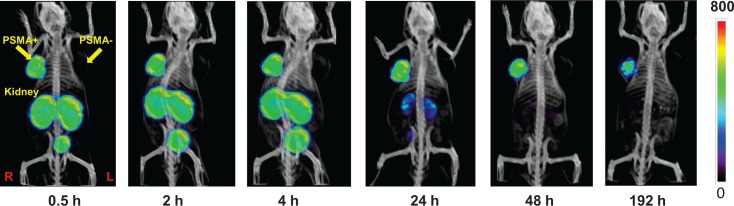
SPECT-CT imaging of [^111^In]3 using PSMA+ PIP and PSMA- flu tumors in a male SCID mouse The mouse was injected intravenously using a single dose of 44.4 MBq (1.2 mCi) of [^111^In]3. Radiochemical uptake was followed up to 192 h post-injection (decay corrected).

**Figure 3:**
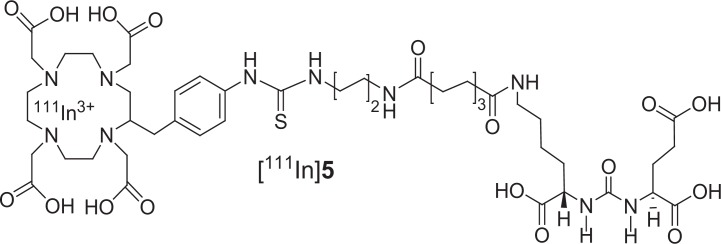
Structure of compound [^111^In]5

Figure 2 shows the pharmacokinetic behavior of [^111^In]3 *in vivo* in SCID mice bearing both PSMA+ PC3-PIP and PSMA- PC3-flu xenografts [26]. We prefer to use the isogenic PSMA+ PIP vs PSMA- flu comparison as the two cell lines are phenotypically identical, differing only in PSMA expression. In this experiment 44.4 MBq (1.2 mCi) of [^111^In]3 was administered intravenously and the animal was imaged repeatedly over an eight day period. Intense radiotracer uptake was seen only in the PSMA+ PIP tumors and in the kidneys. Kidney uptake of the radiotracer is partially due to its route of excretion as well as to specific uptake from the expression of PSMA in mouse kidneys [27]. Clearance of radioactivity from kidney and non-target tissues was more rapid than from target tumor such that by 48 h post-injection (p.i.) a high tumor/background ratio was observed (Figure 2). Significantly, PSMA+ tumor was possible to image out to eight days p.i. To validate the *in vivo* imaging data, [^111^In]3 was also assessed for its pharmacokinetics *ex vivo*. Table 2 shows the percentage injected dose per gram (%ID/g) of radiotracer in selected organs at 2 h and 24 h p.i. Compound [^111^In]3 displayed PSMA-dependent binding in PSMA+ PC3-PIP xenografts with continuous accumulation at the tumor site out to 24 h. We observed tumor uptake values of 31.93 ± 5.87 and 34.03 ±7.53%ID/g (SEM) at 2 and 24 h, respectively. The blood, spleen, gastrointestinal tract, kidney and bladder displayed the highest uptake at 2 h. Steady clearance from the kidneys was demonstrated, from 168.67 ± 14.12 at 2 h to 66.86 ± 14.22%ID/g at 24 h. The tumor uptake values of [^111^In]3 compare favorably with low molecular weight monovalent PSMA imaging agents [10, 13, 14, 16, 22, 28-31], including N-[N-[(S)-1,3-dicarboxypropyl]carbamoyl]-4-[^18^F]fluorobenzyl-L-cysteine, [^18^F]DCFBC [15], which has recently been administered to human subjects [30]. We also compared the *in vivo* properties of the bivalent compound [^111^In]3, with that of one of our lead DOTA-chelated monovalent compounds, [^111^In]5 (Figure 3 and Table 3). The synthesis and characterization of 5 [32] will be published elsewhere. PSMA+ tumor uptake for [^111^In]5 at 2 h p.i. was 29.72 ± 8.09% ID/g, in the same range as that for the bivalent compound [^111^In]3. However at 24 h p.i. monovalent [^111^In]5 showed significantly lower uptake (23.17 ± 3.53% ID/g) than bivalent [^111^In]3 (34.03 ± 7.53%ID/g). At all time points renal retention of [^111^In]5 was significantly lower than that for [^111^In]3. The prolonged tumor retention and rapid clearance from non-target tissues led to very high target to non-target ratios for the bivalent [^111^In]3 at 24 h: PSMA+ PIP to PSMA- flu tumor ratio of 379; tumor to blood ratio of 2,254; and, tumor-to-muscle ratio of 1,220. The corresponding monovalent compound [^111^In]5 demonstrated values of 265, 1,027 and 1,136, in the respective comparisons. The higher uptake and significant retention of [^111^In]3 compared to [^111^In]5 in tumors reflects the advantages of the multimeric design of the former, which affords improved retention *in vivo* in addition to the anticipated multivalent effects on target binding affinity. One explanation for those results could be that the binding of one PSMA-targeting moiety would significantly enhance the local concentration of the other PSMA-targeting moiety of the homodimer in the vicinity of the active site of PSMA, which may lead to a faster rate of receptor binding or a slower rate of dissociation and translate into higher uptake and longer retention time in the tumor. The apparent increase in molecular size may also prolong circulation time of the dimer and consequently reduce the tumor washout rate.

**Table 2:** Biodistribution of [111In]3.*

	2 H	24 H
**blood**	0.12 ± 0.04	0.02 ± 0.01
**heart**	0.16 ± 0.05	0.03 ± 0.01
**lung**	1.84 ± 0.26	0.17 ± 0.04
**liver**	0.19 ± 0.03	0.16 ± 0.03
**stomach**	0.22 ± 0.07	0.03 ± 0.01
**pancreas**	0.43 ± 0.10	0.05 ± 0.02
**spleen**	12.33 ± 3.02	0.64 ± 0.22
**fat**	0.57 ± 0.17	0.19 ± 0.23
**kidney**	168.67 ± 14.18	66.86 ±14.22
**muscle**	0.16 ± 0.08	0.03 ± 0.01
**small intestine**	0.10 ± 0.03	0.04 ± 0.01
**large intestine**	0.27 ± 0.05	0.05 ± 0.03
**bladder**	2.61 ± 1.36	0.52 ± 0.27
**PC-3 PIP**	31.93 ± 5.87	34.03 ± 7.53
**PC-3 flu**	0.16 ± 0.03	0.09 ± 0.03
**PIP:flu**	203	379
**PIP:blood**	257	2,254
**PI:muscle**	199	1,220

* Results are expressed as the percentage injected dose per gram (%ID/g) of tissue; n = 4.

**Table 3:** Biodistribution of [111In]5.*

	2 H	24 H
**blood**	0.28 ± 0.05	0.02 ± 0.01
**heart**	0.16 ± 0.04	0.03 ± 0.01
**lung**	1.12 ± 0.32	0.10 ± 0.02
**liver**	0.25 ± 0.07	0.17 ± 0.02
**stomach**	0.19 ± 0.05	0.04 ± 0.01
**pancreas**	0.24 ± 0.05	0.04 ± 0.01
**spleen**	4.88 ± 2.63	0.32 ± 0.06
**fat**	0.83 ± 0.61	0.02 ± 0.01
**kidney**	110.31± 15.96	7.52 ± 2.38
**muscle**	0.12 ± 0.04	0.02 ± 0.01
**small intestine**	0.17 ± 0.04	0.05 ± 0.01
**large intestine**	0.21 ± 0.07	0.06 ± 0.02
**bladder**	0.91 ± 0.37	0.37 ± 0.16
**PC-3 PIP**	29.72 ± 8.09	23.17 ± 3.53
**PC-3 flu**	0.22 ± 0.05	0.09 ± 0.02
**PIP:flu**	133	264
**PIP:blood**	106	1,027
**PIP:muscle**	242	1,136

* Results are expressed as the percentage injected dose per gram (%ID/g) of tissue; n = 4.

The technique described is able to be generalized to other modalities and for molecular radiotherapy. Since DOTA is a general chelating agent, 3 may also be used with other radiometals such as ^68^Ga, ^64^Cu or ^86^Y for positron emission tomography (PET) or ^90^Y and ^177^Lu for therapy. Technetium-99m can also be incorporated by replacing DOTA with standard peptide-based chelating agents containing nitrogen and sulfur donors (N_3_S, N_2_S_2_), the HYNIC chelator or by use of single amino acid chelating (SAAC) agents [33]. Further attesting to its utility, bivalent 2 can also be used as a versatile intermediate for medically important nonmetals, such as the radiohalogenated imaging isotopes ^18^F, ^123^I or ^211^At/^131^I for radiotherapy. Other fluorophores/chelating agents/radiometals/nonmetals/cytotoxic agent combinations can also be envisioned using this approach. Another significant aspect of the multivalent scaffold is that it will enable us to generate systematically at least 4- and 8-valent urea compounds from the lysine-diamine intermediate 4 upon repeated conjugation of 4 with Fmoc-Lys(Fmoc-OH) to produce a lysine-based multimeric urea dendron.

## METHODS

### Chemistry

Solvents and chemicals obtained from commercial sources were of analytical grade or better and used without further purification. All 9-fluorenylmethyloxycarbonyl (Fmoc) protected amino acids including the Fmoc-Lys(Boc)-Wang resin and benzotriazol-1-yloxy)tripyrrolidinophosphonium hexafluorophosphate (PyBOP) were purchased from Chem Impex International, Inc. (Wooddale, IL). Boc-Lys(Azide)-OH was purchased from Anaspec. Carrier-free [^111^In]InCl_3_ was purchased from MDS Nordion (Ottawa, ON, Canada). DOTA-NHS-ester (1,4,7,10-Tetraazacyclododecane-1,4,7,10-tetraacetic acid mono N-hydroxysuccinimide ester) was purchased from Macrocyclics, Inc. (Dallas, TX). Indium (III) nitrate, 2-ethoxy-1-ethoxycarbonyl-1,2-dihydroquinoline (EEDQ), triethylsilane (Et_3_SiH), N,N-diisopropylethylamine (DIEA) and triethylamine (TEA) were purchased from Sigma-Aldrich (Saint Louis, MO). All other chemicals were purchased from Thermo Fisher Scientific (Pittsburgh, PA) unless otherwise specified. Analytical thin-layer chromatography (TLC) was performed using Aldrich aluminum-backed 0.2 mm silica gel Z19, 329-1 plates and visualized by ultraviolet light (254 nm), I_2_ and 1% ninhydrin in EtOH. Flash chromatography was performed using MP SiliTech 32-63 D 60Å silica gel purchased from Bodman (Aston, PA). All experiments were performed in duplicate or triplicate to ensure reproducibility. ^1^H NMR spectra were recorded on a Bruker UltrashieldTM 400 MHz spectrometer. Chemical shifts (δ) are reported in ppm downfield by reference to proton resonances resulting from incomplete deuteration of the NMR solvent. Low resolution ESI mass spectra were obtained on a Bruker Daltonics Esquire 3000 Plus spectrometer. High resolution mass spectra were obtained by the University of Notre Dame Mass Spectrometry & Proteomics Facility, Notre Dame, IN using ESI by direct infusion on a Bruker micrOTOF-II.

High-performance liquid chromatographic purification of 1 - 3 were performed using a Phenomenex C_18_ Luna 10 × 250 mm^2^ column on a Waters 600E Delta LC system with a Waters 486 variable wavelength UV/Vis detector, both controlled by Empower software. For HPLC purification of radiolabeled [^111^In]3, a Waters Novapak C_18_ 150 × 3.9 mm^2^ column was used. HPLC was performed using the following methods. Method 1: Solvent A (0.1% TFA in water) and solvent B (0.1% TFA in CH_3_CN), flow rate 8 mL/min. The elution gradient was 95% A and 5% B for 5 min and 95% A to 50% A and 5% B to 50% B over 5 - 35 min; Method 2: The elution gradient was 95% A and 5% B for 5 min and 95% A to 70% A and 5% B to 30% B over 5 - 35 min, Method 3: Solvent A (0.1% TFA in water) and solvent B (0.1% TFA in methanol), flow rate 8 mL/min. The elution gradient was 0 - 5 min, 77% A and 23% B for 0 – 5 min, 77% A to 70% A and 23% B to 30% B for 5 - 35 min, and 70% A to 77% A and 30% B to 23% B for 35 min. Method 4: Solvent A (0.1%TFA in water) and solvent B (0.1%TFA in CH_3_CN), flow rate 1 mL/min. The elution gradient was 83% A and 17% B for 0 - 5 min, and 83% A to 70% A and 17% B to 30% B for 5 - 25 min.

#### (3S,7S)-26-Azido-5,13,20-trioxo-4,6,12,21-tetraazahexacosane-1,3,7,22-tetracarboxylic acid, Compound 1

This compound was prepared following our previous report [34]. Briefly, commercially available Boc-Lys(Azide)-OH was treated with a solution of TFA/CH_2_Cl_2_ (1:1) at ambient temperature for 4h to remove the Boc group. After solvent removal, the crude product, H-Lys(azide)-OH, was directly used for the next step. To a solution of H-Lys(azide)-OH (50 mg, 0.29 mmol in 500 μL DMSO) was added NHS-ester of Lys-Glu urea (24 mg, 0.43 mmol in 500 μL DMSO) [16] and DIEA (100 μL) and left at ambient temperature for 4 h. Solvent was evaporated to dryness and the residue was dissolved in water and purified by HPLC (Method 1). Retention time (R_t_): 17.1 min. ^1^H NMR (δ, ppm, DMSO): 8.06 (m, 2H), 7.74 (m, 2H), 6.34-6.29 (m, 2H), 4.16-4.03(m, 3H), 3.00 (m, 2H), 2.33-1.27(m, 28H). ESI-MS m/Z: 630 [M+H]^+^.

#### 6-amino-2-(2,6-dipropiolamidohexanamido)hexanoic acid, 4

Compound 4 was synthesized using standard Fmoc mediated solid phase peptide synthesis. Formation and masking of free amines was assessed using the Kaiser test [35]. Washing the resin with 3 mL DMF three times, 1 minute each, before and after each step was performed until liberation of the final product from the resin. All steps were performed at ambient temperature unless otherwise mentioned. After being swollen by 3 mL DMF, Fmoc-Lys- (Boc)-Wang resin (500 mg, 0.18 mM) was deprotected by settling with 3 mL 20% piperidine in DMF x 2, 5 min each time, before coupling with Fmoc-Lys-(Fmoc)-OH (318 mg, 0.54 mM) preactivated with DIEA (124 uL, 0.72 mM) and PyBOP (280 mg, 0.54 mM in 3 mL DMF). The last coupling was performed twice, 30 minutes in duration each time. The Fmoc groups were removed using 3 mL 20% piperidine in DMF x 2, 5 min each time. Coupling with propiolic acid (75 mg, 1.08 mM) was achieved using a solution of EEDQ (268 mg, 1.08 mM) as a coupling reagent in 2 mL DMF and accelerated *via* exposure to microwave irradiation x 5, 30 sec each time. Ten percent of the maximum power of a standard kitchen microwave was enough to achieve complete coupling as indicated by a negative Kaiser test [35]. Cleavage of 4 from the resin was achieved by settling with 2 mL TFA/H_2_O/TES (95/2.5/2.5) mixture for 30 min followed by washing twice with 2 mL 100% TFA. The collected fractions were evaporated under vacuum after which the concentrated product was purified using a Sep-Pak® Vac 35 cc tC_18_ tube (Waters, WAT043350). Compound 4 was eluted with 5% acetonitrile in water (0.1 % TFA). HPLC: Method 2, R_t_: 10 min. ^1^H NMR (DMSO-*d*_6_) (δ, ppm): 8.89 (m, 1H) 8.72 (m, 1H), 8.21 (m, 2H), 7.73 (m, 2H), 4.23 (m, 1H) 4.16-4.10 (m, 4H), 3.04 (m, 2H), 2.77 (m, 2H), 1.74-1.27 (m, 12H). ESIMS m/Z: 379 [M+H]^+^.

#### (7S)-26-(4-((1-((5-amino-1-carboxypentyl)amino)-1-oxo-6-(1-((7S)-1,3,7,22-tetracarboxy-5,13,20-trioxo-4,6,12,21-tetraazahexacosan-26-yl)-1H-1,2,3-triazole-4-carboxamido)hexan-2-yl)carbamoyl)-1H-1,2,3-triazol-5-yl)-5,13,20-trioxo-4,6,12,21-tetraazahexacosane-1,3,7,22-tetracarboxylic acid, PSMA-targeted homobivalent compound 2

Compound 1 (49 mg, 76.7 μM) and 4 (0.5 eq, 14.5 mg, 38.3 μM) were dissolved in 1 mL H_2_O/*t*-BuOH (1/1). To that solution, sodium ascorbate (6 mg, 30 μM) and Cu(OAc)2 (3 mg, 15 μM) were added consecutively, the mixture was purged with N_2_ gas and stirred at ambient temperature overnight. Compound 2 was purified using C_18_ SepPak® Vac 2 g column through which the product was successfully eluted using 70/30 water/acetonitrile (0.1% TFA). Compound 2 was further purified by HPLC (Method 1). R_t_: 13.9 min. ESI-MS m/Z: 1638 [M+H]^+^.

#### (7S)-26-(4-((1-((1-carboxy-5-(2-(4,7,10-tris(carboxymethyl)-1,4,7,10-tetraazacyclododecan-1-yl)acetamido)pentyl)amino)-1-oxo-6-(1-((7S)-1,3,7,22-tetracarboxy-5,13,20-trioxo-4,6,12,21-tetraazahexacosan-26-yl)-1H-1,2,3-triazole-4-carboxamido)hexan-2-yl)carbamoyl)-1H-1,2,3-triazol-5-yl)-5,13,20-trioxo-4,6,12,21-tetraazahexacosane-1,3,7,22-tetracarboxylic acid, DOTA conjugated PSMA- targeted homobivalent compound 3

To a solution of 2 (4 mg, 2.44 μM in 500 μL DMF) was added DOTA-NHS ester (1.5 mg, 3.66 μM in 500 μL DML) and DIEA (50 μL) and left at ambient temperature for 4h. Solvent was removed under vacuum and the residue was dissolved in 2 mL water and was purified using HPLC Method 3. R_t_: 26.2 min ESIMS m/Z: 2024[M+H]^+^, HRESI-MS (m/z): HRESI-MS m/Z: Calcd. for C_86_H_135_N_22_O_34_, 2023.9824; Found 2023.9820.

**[^113^/^115^In]3**

To a solution of In(NO_3_)_3_ (1 mg, 20 μmol in 100 μL) in deionized water was added 3 (1 mg, 0.48 μmol) in 500 μL 0.3 M NaOAc. The resulting solution was heated at 95°C for 1 h. The solution was purified by HPLC Method 3. The retention time for the product was at 25.8 min. Yield: ~ 50%. ESIMS m/Z: 1067 [M+H]^2+^.

**[^111^In]3**

For each radiolabeling reaction, approximately 50 - 70 μg of 3 in 300 mM NaOAc (purged under N_2_ for 2-3 min) was incubated with 111-148 MBq (3 - 4 mCi) ^111^InCl_3_ at pH 5.5 - 6 for 20 h at 95°C. The reaction solution was diluted with 1 mL water. Complexation was monitored by injecting aliquots of 20 - 40 μL of the solution onto the HPLC. The radiolabeled product [^111^In]3 was obtained in ~ 70 - 90% radiochemical yield and the radiochemical purity was > 98% as measured by ITLC (Gelman ITLC strips, 10mM EDTA). HPLC Method 4 was used to purify the radiolabeled product [^111^In]3. R_t_: 13.5 min for the desired product and R_t_: 15.4 min for the free ligand. The specific radioactivity of the agent was ~ 8.4 – 204.4 GB/μmol. The acidic eluate was neutralized with 100 μL of PBS solution and the volume of the eluate was reduced under vacuum to dryness. The solid residue was diluted with saline to the desired radioactivity concentration for biodistribution and imaging studies.

### Biological Studies

#### PSMA Inhibition

The PSMA inhibitory activities of 1 - 3 and [^113/115^In]3 were determined using a fluorescence-based assay according to a previously reported procedure [8]. Briefly, lysates of LNCaP cell extracts (25 μL) were incubated with the inhibitor (12.5 μL) in the presence of 4 μM NAAG (12.5 μL) for 120 min. The amount of glutamate released upon hydrolysis of NAAG was measured by incubating with a working solution (50 μL) of the Amplex Red Glutamic Acid Kit (Life Technologies, Grand Island, NY) for 60 min. Fluorescence was measured with a VICTOR3V multilabel plate reader (Perkin Elmer Inc., Waltham, MA) with excitation at 530 nm and emission at 560 nm. Inhibition curves were determined using semi-log plots and IC_50_ values were determined at the concentration at which enzyme activity was inhibited by 50%. Assays were performed in triplicate. Enzyme inhibitory constants (*K*_i_ values) were generated using the Cheng-Prusoff conversion [36]. Data analysis was performed using GraphPad Prism version 4.00 for Windows (GraphPad Software, San Diego, CA).

#### Cell Culture and Animal Models

Sublines of the androgen independent PC-3 human prostate cancer xenograft originally derived from an advanced androgen independent bone metastasis were used. Those sublines have been modified to express high (PC-3 PIP) and low (PC-3 flu) PSMA levels and were generously provided by Dr. Warren Heston (Cleveland Clinic). Both PSMA-expressing (PC-3 PIP) and non-expressing (PC-3 flu) prostate cancer cell lines were grown in RPMI 1640 medium (Mediatech Inc., Manassas, VA) containing 10% fetal bovine serum (FBS) (Sigma Aldrich, St.Louis, MO) and 1% Pen-Strep (Mediatech Inc., Manassas, VA) as previously described [12]. All cell cultures were maintained at 5% carbon dioxide (CO_2_), at 37°C in a humidified incubator. Animal studies were carried out in full compliance with the regulations of the Johns Hopkins Animal Care and Use Committee. Six- to eight-week-old male, non-obese diabetic (NOD)/severe-combined immunodeficient (SCID) mice (Johns Hopkins Animal Core, Baltimore, MD) were implanted subcutaneously (s.c.) with PC-3 PIP (PSMA+) and PC-3 flu (PSMA-) cells (2 × 10^6^ in 100 μL of Matrigel) at the forward right and left flanks, respectively. Mice were imaged or used in biodistribution assays when the xenografts reached 5 to 7 mm in diameter.

#### Gamma Scintigraphy and SPECT/CT

Compound [^111^In]3 was imaged using male SCID mice. Xenograft models were generated as described above. Mice were anesthetized using 1% isoflurane in oxygen flowing at 0.6 L/min prior to and during radiochemical injection. Mice were injected *via* the tail vein with approximately 1.2 mCi (44.4 MBq) of [^111^In]3 formulated in 100 μL of saline, pH 7. After allowing for 30 - 60 min of radiochemical uptake, anesthetized mice were placed on the scanner gantry and secured with medical tape while the anesthetic flow was increased to 0.8 L/min. The body temperature of the mice was maintained by covering them with several layers of absorbent, disposable pads and illumination with a dissection lamp during scanning. Single-pinhole median-energy (PHME) collimators with an aperture size of 1.0 mm, and stepwise rotation for 64 projection angles in a full 360° rotation, 40 s increments were used for SPECT imaging. The radius of rotation (ROR) was set at 7 cm, which provided a field of view of 7.5 cm to cover the mouse body from head to bladder. A CT scan was performed prior to scintigraphy for both anatomical co-registration and attenuation correction. A total of 512 projections were acquired in a 2 min continuous rotation mode covering a full 360° rotation. Data were reconstructed and fused using commercial software from the vendor (Gamma Medica-Ideas, Northridge, CA), which includes a 2D-OSEM algorithm. Data were analyzed and volume-rendered images were generated using AMIDE software (SourceForge, http://amide.sourceforge.net/).

#### Biodistribution

PSMA+ PC-3 PIP and PSMA- PC-3 flu xenograft-bearing SCID mice were injected *via* the tail vein with 0.74 MBq (20 μCi) of [^111^In]3. Four mice were sacrificed by cervical dislocation at 2 and 24 h p.i. The heart, lungs, liver, stomach, pancreas, spleen, fat, kidney, muscle, small and large intestines, urinary bladder, and PC-3 PIP and flu tumors were quickly removed. A 0.1 mL sample of blood was also collected. Each organ was weighed, and the tissue radioactivity was measured with an automated gamma counter (1282 Compugamma CS, Pharmacia/LKB Nuclear, Inc., Gaithersburg, MD). The percentage injected dose per gram of tissue (%ID/g) was calculated by comparison with a standard dilution of the initial dose. All measurements were corrected for radioactive decay.
